# *Aspidosperma pyrifolium*, a medicinal plant from the Brazilian caatinga, displays a high antiplasmodial activity and low cytotoxicity

**DOI:** 10.1186/s12936-018-2568-y

**Published:** 2018-11-26

**Authors:** Isabela P. Ceravolo, Carlos L. Zani, Flávio J. B. Figueiredo, Markus Kohlhoff, Antônio E. G. Santana, Antoniana U. Krettli

**Affiliations:** 1 Experimental and Human Malaria Section, Instituto René Rachou, FIOCRUZ, Belo Horizonte, MG 30.190-009 Brazil; 2 Chemistry of Natural Products Section, Instituto René Rachou, FIOCRUZ, Belo Horizonte, MG 30.190-009 Brazil; 3 Centro de Ciências Agrárias, Universidade Federal de Alagoas, Maceió, AL 57072-900 Brazil

**Keywords:** *Aspidosperma pyrifolium*, *Plasmodium falciparum*, Ethnopharmacology, Medicinal plants, Anti-malarial

## Abstract

**Background:**

Several species of *Aspidosperma* plants are referred to as remedies for the treatment of malaria, especially *Aspidosperma nitidum*. *Aspidosperma pyrifolium*, also a medicinal plant, is used as a natural anti-inflammatory. Its fractionated extracts were assayed in vitro for activity against malaria parasites and for cytotoxicity.

**Methods:**

*Aspidosperma pyrifolium* activity was evaluated against *Plasmodium falciparum* using extracts in vitro. Toxicity towards human hepatoma cells, monkey kidney cells or human monocytes freshly isolated from peripheral blood was also assessed. Anti-malarial activity of selected extracts and fractions that presented in vitro activity were tested in mice with a *Plasmodium berghei* blood-induced infection.

**Results:**

The crude stem bark extract and the alkaloid-rich and ethyl acetate fractions from stem extract showed in vitro activity. None of the crude extracts or fractions was cytotoxic to normal monkey kidney and to a human hepatoma cell lines, or human peripheral blood mononuclear cells; the MDL_50_ values of all the crude bark extracts and fractions were similar or better when tested on normal cells, with the exception of organic and alkaloidic-rich fractions from stem extract. Two extracts and two fractions tested in vivo caused a significant reduction of *P. berghei* parasitaemia in experimentally infected mice.

**Conclusion:**

Considering the high therapeutic index of the alkaloidic-rich fraction from stem extract of *A. pyrifolium*, it makes the species a candidate for further investigation aiming to produce a new anti-malarial, especially considering that the active extract has no toxicity, i.e., no mutagenic effects in the genototoxicity assays, and that it has an in vivo anti-malarial effect. In its UPLC-HRMS analysis this fraction was shown to have two major components compatible with the bisindole alkaloid Leucoridine B, and a novel compound, which is likely to be responsible for the activity against malaria parasites demonstrated in in vitro tests.

## Background

Resistance of *Plasmodium* parasites to classical drugs unfortunately now includes artemisinin derivatives, the latest weapon to fight malaria in areas of drug resistance [1]. First isolated from the Chinese medicinal plant *Artemisia annua* (sweet wormwood), artemisinin derivatives have a potent effect against drug-resistant parasites [2]. New drugs to fight malaria are needed due to the spread of *Plasmodium falciparum* resistant to available anti-malarial drugs [1, 3]. *Plasmodium vivax*, the species most prevalent in South America, also shows resistance to chloroquine, making malaria control more difficult [4–6].

The use of plants as medicines, a millennial tradition in Asian and African countries, has become common in the Western world as well [7–9]. One-third of adults use herbs as alternative therapy in their primary forms, or as plant mixtures claimed to be non-toxic even after long-term use [10]. Herbal remedies remain important to control malaria in poor, endemic areas [11–15].

Various medicinal plant species used in Latin American countries against fever and/or malaria proved to be active when tested in vitro against malaria parasites and/or in experimentally infected mice [15–24]. Among them are the plants of the Apocynaceae family, rich in monoterpene indole alkaloids, and active in vitro against *P. falciparum* [25–32]. The *Aspidosperma nitidum* (Apocynaceae) stem bark used to treat fever and malaria in the Amazon region was active at low concentrations against *P. falciparum* and in mice infected with sensitive *Plasmodium berghei* parasites [29].

The species *Aspidosperma pyrifolium*, known in Brazil as *pereiro*, *pereiro*-*preto* or *pereiro*-*do*-*sertão* [33], grows widely in the Brazilian *caatinga*. Here, this species was submitted to chemical fractionation followed by biological tests against *P. falciparum* in vitro and *P. berghei* in mice, in parallel with tests of cytotoxicity in vitro.

## Methods

### Plant material

Plant collection and access to genetic resources were approved by CNPq (Process N°010861/2013-0) and registered in the National System for the Management of Genetic Heritage and Associated Traditional Knowledge (SisGen, Process N°A61DDB0 and N°A646A52, respectively).

**Fig. 1:**
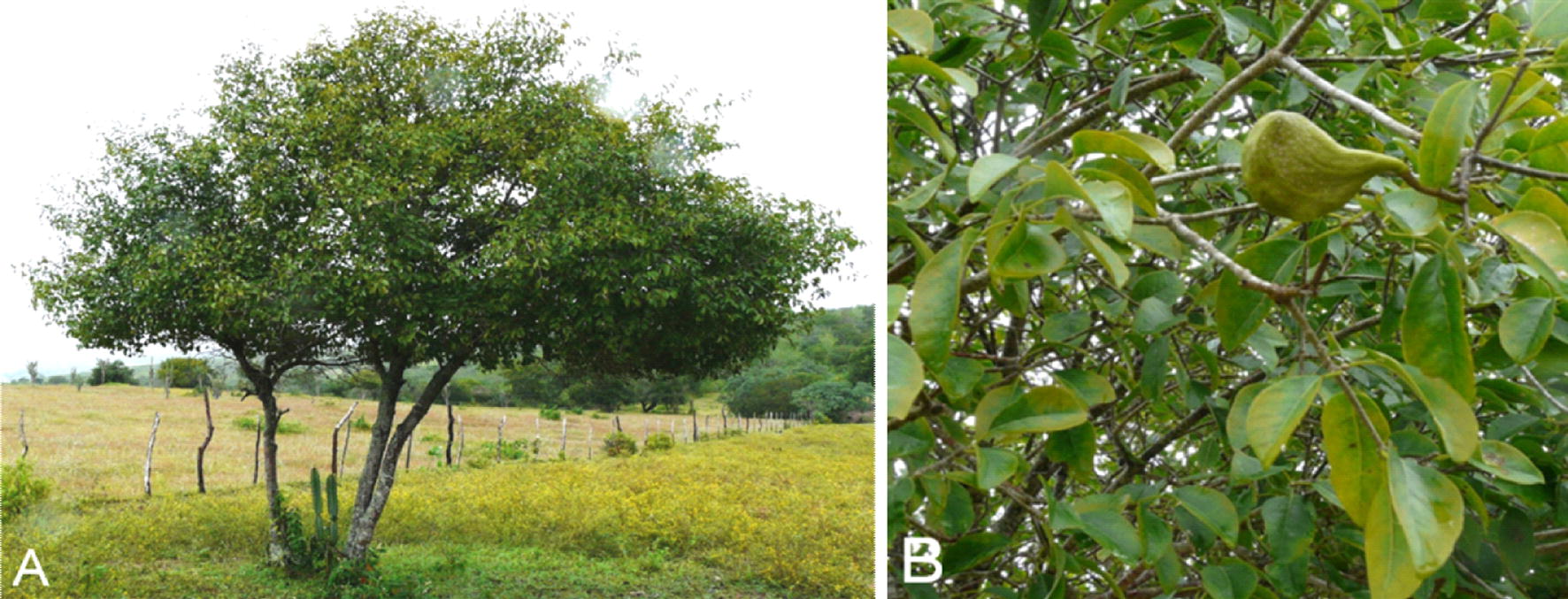
*Aspidosperma pyrifolium*, a medicinal plant used as anti-inflammatory, known as *pereiro* in Brazil. **A** adult tree; **B** parts used for classification

Parts of *A. pyrifolium* (Fig. 1) were collected in São José da Tapera (Alagoas, Brazil) in October 2001; the leaves were collected in 2016 at the margins of São Francisco River. The species was identified by José Elias de Paula, from the Department of Botany (Universidade de Brasília, UNB), where a plant voucher is deposited (JEP 3686-UnB).

### Extract preparation and fractionation

**Fig. 2:**
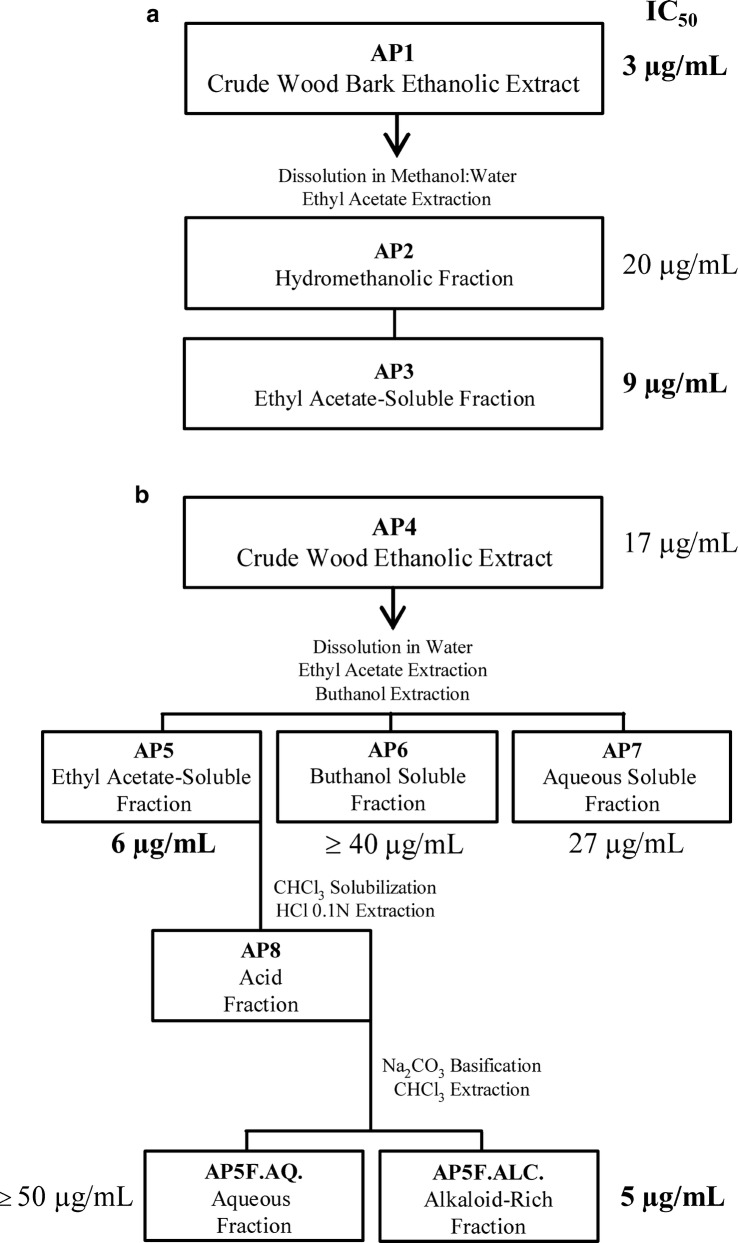
Fractionation workflow of the plant stem bark (**a**), and stem (**b**) extracts from *Aspidosperma pyrifolium*. The in vitro activity against *P. falciparum* (IC_50_) highlighted in bold represents the best results

Purification of *A. pyrifolium* crude stem bark extract was performed as previously described [33] (Fig. 2a). Briefly, the stem bark of the plant was air-dried at room temperature and ground to a course powder (2.5-mm mesh size) using a laboratory mill. The powdered material (3.0 kg) was extracted with 95% ethanol (5.5 l) in a Soxlet apparatus for 72 h, and then concentrated under reduced pressure in a rotary evaporator. The remaining water was removed using a freeze dryer yielding 147 g of crude stem bark extract (AP1) and kept at 4 °C until use. AP1 was dissolved in methanol, water was added (2:3) and the mixture was partitioned with ethyl acetate. The hydromethanolic phase was lyophilized producing 55 g of an alkaloid rich fraction (AP2), and the ethyl acetate phase was concentrated under pressure yielding 91 g of the AP3 fraction. The alkaloids were detected in silica gel TLC plates by spraying with Dragendorff reagent.

The fractionation procedures of the stem extract are summarized in Fig. 2b. The isolated plant material was air-dried as described above; the powder was extracted with 95% ethanol and concentrated under reduced pressure yielding 147 g of extract (AP4). This extract was dissolved in water: methanol (2:3), and the mixture partitioned with ethyl acetate. The organic fraction (AP5) was solubilized in chloroform and partitioned with hydrochloric acid 0.1 M. The aqueous acidic phase was separated and adjusted to pH 10 with 1 M NaOH. The free base alkaloids were extracted with chloroform, producing an aqueous (AP5F.AQ) and an alkaloid-rich (AP5F.ALC) fraction. The aqueous phase was partitioned with butanol yielding an organic (AP6) and an aqueous (AP7) fraction. Dragendorff reagent was used to reveal the presence of alkaloids in TLC plates. The extracts and fractions obtained were further used in biological assays. Fractionation of the crude leaves, root and root bark extracts was as described above for the stem.

### Ultra-performance chromatographic coupled to mass spectrometry (LC–MS/MS)

The analyses were performed on a Nexera UHPLC-system (Shimadzu) hyphenated to a maXis ETD high-resolution ESI-QTOF mass spectrometer (Bruker) controlled by the Compass 1.5 software package (Bruker). Samples were diluted to final concentration of 5 mg/ml and 1 µl was injected on a Shimadzu Shim-Pack XR-ODS-III column (C18, 2.2 µm, 2.0 × 150 mm) at 40°C at a flow rate of 400 μl/min. An additional identical run was performed with 5 µl (50 µg) injected, with 200 µl fractions collected in each well of a microtitre plate. The mobile phases A and B (0.1% formic acid in distilled water and acetonitrile, respectively) were used to form an isocratic run of 5% B during the initial 5 min, followed by a linear gradient to 100% B in 40 min and a hold at 100% B for 5 min. The mass spectra were acquired in positive mode at a spectra rate of 2 Hz. Ion-source parameters were set to 500 V end plate offset, 4500 V capillary voltage, 3.0 bar nebulizer pressure, 8 l/min and 200 °C dry gas flow and temperature respectively. Data-dependent fragment spectra were recorded using a collision energy range between 15 and 60 eV. Ion cooler settings were optimized for an m/z 40–1000 range using a calibrant solution of 1 mM sodium formate in 50% 2-propanol. Mass calibration was achieved by initial ion-source infusion of 20 μl calibrant solution and post-acquisition recalibration of the raw data. Compound detection was performed by chromatographic peak dissection with subsequent formula determination according to exact mass and isotope pattern (MS1). Identification was based on comparison of compound fragment spectra (MS2) with reference spectra of an in-house database of standard compounds, the public spectra database MassBank [34], as well as in silico fragment spectra generated from the Universal Natural Product Database (UNPD-ISDB) [35]. The solvents were removed in a vacuum centrifuge at 45 °C before the *P. falciparum* assay described below.

### Continuous cultures of *Plasmodium falciparum*

The blood forms of *P. falciparum* (W2 clone, chloroquine-resistant and mefloquine-sensitive) [36] were cultured as described [37]. After synchronization with sorbitol, the ring forms parasitized erythrocytes were used in tests of anti-parasite activity using the immune enzymatic assay with specific monoclonal antibodies to a parasite protein rich in histidine and alanine (HRPII), as described [38]. The antibodies were commercially acquired (MPFM55A and MPFM55P, ICLLAB, Portland, OR, USA). Endotoxin-free sterile disposables labware were used in all experiments.

The half-maximal drug inhibitory concentration (IC_50_) was estimated by curve fitting with the software Origin (OriginLab Corporation, USA) compared to parasite growth in drug-free medium. Fractions with IC_50_ values bellow 10 µg/ml were considered active; between 10 e 20 µg/ml as partially active, and above 20 µg/ml as inactive as demonstrated before [30, 31]. The chloroquine was used as anti-malarial control in all assays.

### Cytotoxicity tests

The toxicity tests of plant extracts and fractions were performed against a monkey kidney cell line (BGM), a human hepatoma cell line (HepG2), and freshly isolated human peripheral blood mononuclear cells (PBMC) collected from healthy volunteers (approved by Ethics Committee, *Centro de Pesquisas René Rachou*-FIOCRUZ, CAAE 03209212.7.0000.5091 at 10/03/2012). Cytotoxicity was evaluated by the MTT assay [(3-(4,5-dimethylthiazol-2-yl)-2,2-diphenyltetrazolium)] as described [39]. The cell viability was expressed as the percentage of the control absorbance in the untreated cells after subtracting the background. The minimum lethal dose for 50% of the cells (MLD_50_) was determined as previously described [40]. The ratio between drug cytotoxicity (MLD_50_) and activity (IC_50_) was used to estimate the selective index (SI), or therapeutic activity as described for *A. nitidum* [29]. A SI ≤ 10 was indicative of toxicity.

### Anti-malarial tests in vivo

Swiss outbred adult female mice (20 ± 2 g) were each inoculated intraperitoneally with 1 × 10^5^ *P. berghei* (strain NK65)-infected red blood cells, as described before [29]. The mice were divided randomly in groups of 3–5 animals per cage and then treated by gavage for 3 consecutive days with 100 mg/kg of the fractions dissolved in DMSO 3% (v/v). Blood samples were collected from the mice tails on day 5 and 10 of infection, fixed with methanol, Giemsa stained and used for parasitaemia determination by microscopy. The extracts and fractions were evaluated in one test and the per cent reduction of parasitaemia calculated considering untreated mice parasitaemia as 100%, the leaves extract was tested three times. Drugs reducing parasitaemia by 40% or more were considered active; reductions between 20 and 40% partially active, and reductions below 20% inactive as demonstrated before [29]. Mice mortality was monitored daily until day 30 post-infection. The chloroquine was used as anti-malarial control at a sub-curative dose of 20 mg/kg. The protocol for animal use was approved by the Ethics Committee at FIOCRUZ (CEUA LW-23/13 at 05/20/2013).

### Statistical analysis

Statistical analysis was performed using Prism 5.0 (GraphPad Software, Inc., La Jolla, CA, USA). The survival of the treated mice when compared to control mice control was analysed using the Kruskal–Wallis rank sum test. Statistical significance was defined as *P* values ≤ 0.05.

### Mutageniticity and genotoxicity tests

The potential of the active alkaloid fraction from the plant stem to induce mutagenic and genotoxic effects in vitro was evaluated with Ames tests [41] performed at the Genotox-Royal Institute (Rio Grande do Sul, Brazil), contract number GT00748. Five strains of *Salmonella typhimurium* with several specially constructed mutants were tested in the presence of various drug concentrations in the absence and presence of the metabolizing rat liver fraction for the ability of a given extract or fraction to induce mutations.

## Results

### In vitro assays

The *A. pyrifolium* extracts and fractions were not toxic at the highest doses tested against the BGM cells (MLD_50_ ≥ 1000 µg/ml), except for the crude extracts from root bark and leaves (MLD_50_ = 287 to 486 µg/ml). Some toxicity to HepG2 cells and to PBMC freshly collected also occurred with most extracts and fractions of the crude stem and stem extracts (Table 1).

**Table 1:** Cytotoxicity (MLD_50_) and activity (IC_50_) in vitro against *Plasmodium falciparum* of *Aspidosperma pyrifolium* extracts and fractions

Plant material (code)^a^	MLD_50_ (µg/ml)^b^			IC_50_^c^ (µg/ml)	Activity
	BGM	HepG2	PBMC		
Crude extracts					
Stem bark (AP1)	≥ 1000	410 ± 67	≥ 1000	3 ± 3	AT
Stem (AP4)	≥ 1000	415 ± 17	404 ± 42	17 ± 4	PA
Root bark (AP9)	287 ± 35	407 ± 60	492 ± 75	14 ± 1	PA
Roots (AP12)	≥ 1000	415 ± 17	404 ± 42	18 ± 5	PA
Leaves	486 ± 15	449 ± 49	548 ± 22	12 ± 4	PA
Fractions from AP1					
Organic (ethyl acetate) (AP3)	≥ 1000	489 ± 13	435 ± 83	9 ± 3	AT
Aqueous (AP2)	≥ 1000	636 ± 55	≥ 1000	20 ± 6	PA
Fractions from AP4					
Organic (ethyl acetate) (AP5)	≥ 1000	316 ± 42	153 ± 13	6 ± 1	AT
Alkaloid-rich (AP5F.ALC)	≥ 1000	418 ± 7	145 ± 17	5 ± 3	AT
Aqueous (AP5F.AQ)	≥ 1000	≥ 1000	≥ 1000	≥ 50	IN
Butanolic (AP6)	≥ 1000	≥ 1000	≥ 1000	≥ 40	IN
Aqueous (AP7)	≥ 1000	≥ 1000	≥ 1000	27 ± 3	IN
Chloroquine	457 ± 22	398 ± 12	150 ± 53	0.07 ± 0.02	AT

*AT* active, *PA* partially active, *IN* inactive

^a^The fractionation steps are summarized in Fig. 2

^b^MLD for 50% of hepatoma cells (HepG2), monkey kidney cells (BGM) or freshly isolated human peripheral blood mononuclear cells (PBMC) in three or four tests with MTT

^c^IC_50_ correspond to the concentration inhibiting 50% growth of blood forms of *P. falciparum* (W2 clone, chloroquine-resistant) in 3–5 independent assays

Among 5 crude extracts (from stem, roots, leaves) and 7 different fractions tested against *P. falciparum* chloroquine-resistant parasites, the best in vitro activity was shown by the crude stem bark extract (AP1) (IC_50_ = 3 µg/ml). This was followed by the alkaloid fraction AP5F.ALC (IC_50_ = 5 µg/ml) and the organic fraction (AP5) (IC_50_ = 6 µg/ml) both from the crude stem extract. The stem (AP4), root bark (AP9), roots (AP12) and leaves extracts, and the aqueous fraction (AP2) from stem bark extract were all partially active; all other fractions were inactive (Table 1). Chloroquine was used as positive control in all assays.

None of the crude extracts or fractions was cytotoxic to HepG2, BGM or human peripheral blood mononuclear cells; the MDL_50_ values of all the crude bark extracts and fractions were similar or better when tested on normal cells, with the exception of AP5 and AP5F.ALC fractions (Table 1).

The ratio between in vitro cytotoxicity and activity or selectivity index (SI) was highest for the crude stem bark (AP1) regardless of the source of cells used to evaluate toxicity in vitro, varying from 137 to 333 (Table 2). The organic (ethyl acetate) fractions from stem bark (AP3), stem (AP5), and the alkaloid-rich fraction (AP5F.ALC) from stem extract (AP4) showed toxicity to the BGM cell line only, with high specific activity to *P. falciparum* reflected in SI values of 111 (AP3), 167 (AP5) and 200 (AP5F.ALC).

**Table 2:** Selectivity indexes of extracts and fractions of *Aspidosperma pyrifolium* to three types of cells

Plant extract and fractions (code)	SI (MLD_50_/IC_50_)^a^		
	HepG2	BGM	PBMC
*Stem bark (AP1)*	137	333	333
Aqueous (AP2)	32	50	50
Organic (ethyl acetate) (AP3)	54	111	48
*Stem (AP4)*	24	59	24
Organic (ethyl acetate) (AP5)	53	167	26
Alkaloid-rich (AP5F.ALC)	84	200	29
*Root bark (AP9)*	29	21	21
*Root (AP12)*	23	56	22
*Leaves*	41	37	46

^a^MLD_50_ and IC_50_ are shown in Table 1. SI was calculated only for the active samples

When evaluated by the Ames test, the alkaloidic-rich fraction (AP5FALC) did not induce reverse mutation in the TA100, TA98, TA97a, TA102 and TA1535 strains of *S. typhimurium* up to the concentration of 40 μg/plate tested in the absence and up to the concentration of 5000 μg/plate in the presence of liver metabolizing fraction of mouse, induced by Aroclor 1254 (-S9).

### Anti-malarial tests

The extracts from the root bark (AP9), root (AP12) and leaves, which were available in sufficient amounts, were also evaluated in mice for their anti-malarial activity. Oral administration (100 mg/kg) of the crude root extract (AP12) for 3 consecutive days reduced *P. berghei* parasitaemia by 75 and 52% on the 5th and 10th days of infection, respectively (Table 3), and by 79% when animals were treated by the crude root bark extract (AP9) on the 5th day. The leaves extract resulted on slightly reduced parasitaemia. The aqueous fraction AP2 (derived from AP1) caused a 93 and 57% reduction on 5th and 10th days of infection; the alkaloid fraction AP5F.ALC (derived from AP4) caused 79 and 57% reduction at both time points. Chloroquine, the standard anti-malarial, was tested in parallel each time at 20 mg/kg. Other samples that presented activity in vitro were not tested in vivo due to insufficient mass amounts available.

**Table 3:** Anti-malarial activity of *Aspidosperma pyrifolium* against *Plasmodium berghei* in mice

Crude extracts and fractions (code)	% Reduction^a^	
	Day 5	Day 10
Extracts		
Root bark (AP9)	79	29
Root (AP12)	75	52
Leaves	23	40
Fractions		
Aqueous (AP2)	93	57
Organic (ethyl acetate) (AP3)	0	34
Alkaloid-rich (AP5F.ALC)	79	57
Chloroquine^b^	100	100

^a^Reduction in parasitaemia compared to control infected non-treated mice; > 40% = active; 20–40% partially active; < 20% inactive. Results from one test, with exception of leaves extract that was tested three times

^b^Chloroquine was tested at 20 mg/kg

### LC–MS/MS analysis

**Fig. 3:**
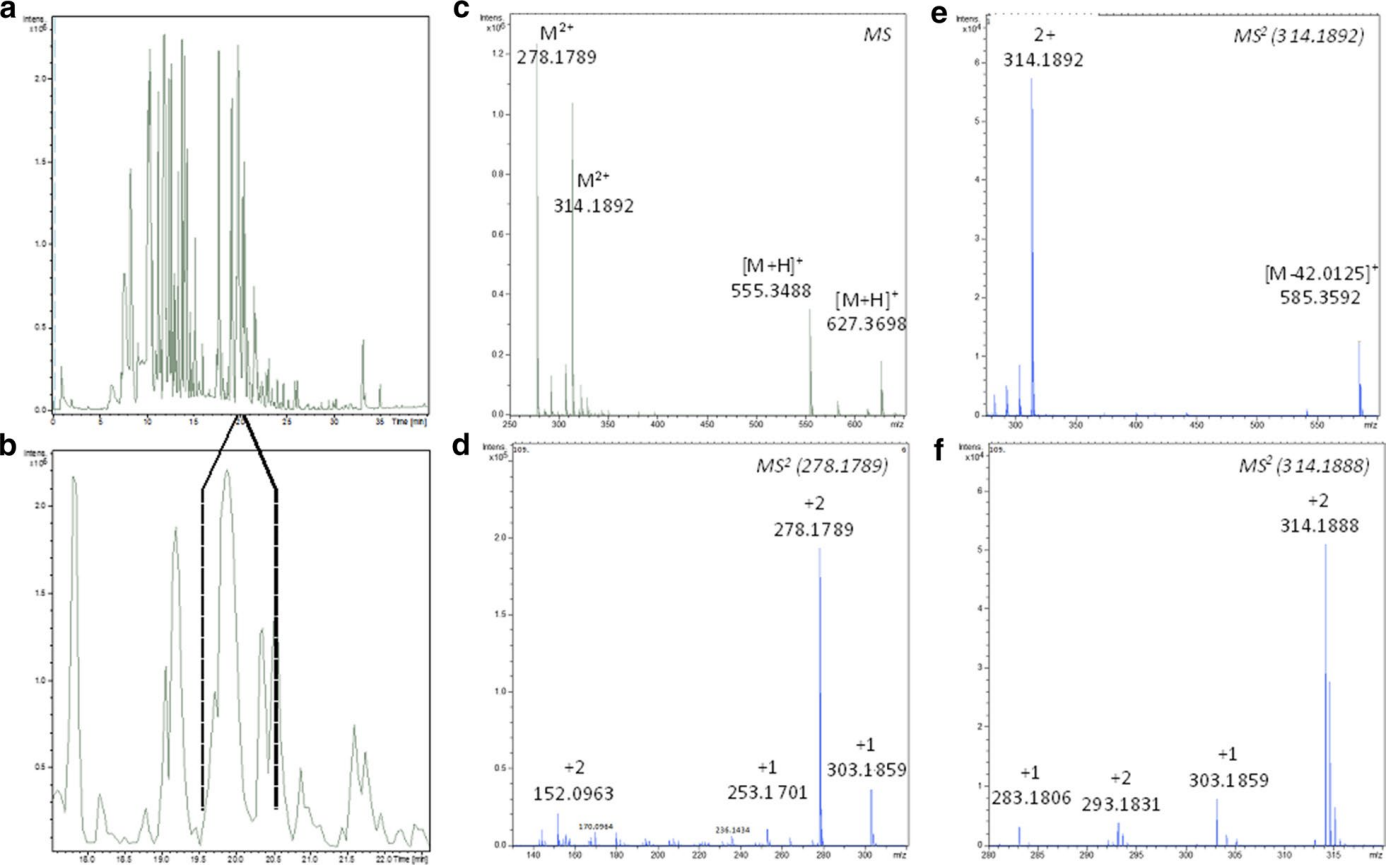
Data generated by LC–MS/MS analysis of the *Aspidosperma pyrifolium* alkaloid fraction. **a** Full chromatogram of AP5-Falc; **b** detail showing peaks of the active fractions (19.5–20.5 min); **c** principal ions detected in the active fractions; **d** MS2 of the double-charged ion 278.1789; **e** MS2 of the double-charged ion 314.1892; **f** detail of the region m/z 280–320 of E

The remaining few milligrams of AP5F.ALC were analysed on an UPLC coupled with a HRMSMS equipment. The fractions collected after the injection of 50 mg in a reversed phase analytical column were assayed in vitro against *P. falciparum.* The two fractions that eluted between 19.5 and 20.5 min (Fig. 3a, b) were active in the bioassay. The *m/z* profile of this region (Fig. 3c) showed that these fractions were composed of two major components, **A** and **B**, exhibiting [M+H]^+^ ions with *m/z* of 555.3488 and 627.3698, compatible with molecular formulas C_38_H_42_N_4_ and C_41_H_46_N_4_O_2_, respectively.

## Discussion

Herbal remedies and medicinal plants are important in remote areas where poor communities have little access to effective anti-malarial drugs [11]. Plants with confirmed biological activity and standardized crude herbal medicines used alone or combined with synthetic drugs represent complementary treatments and may help to inhibit the development of drug resistant parasites [42].

The *A. nitidum* species is popularly used to treat fever and/or malaria in the Brazilian Amazon [29]; various species of *Aspidosperma* have anti-*P. falciparum* activity [13, 25, 27, 29–32, 43]. *Aspidosperma pyrifolium* is used in some areas to treat inflammation of the urinary tract and dermatitis [44]; the anti-inflammatory effect of their seeds was also observed in a model of Parkinson’s disease [45], but not as an anti-malarial, although alkaloids isolated from *A. pyrifolium* have already demonstrated antiplasmodial activity [46]. Such effect is herein demonstrated for the first time, together with low toxicity to cell lines and to fresh human PBMC. This is an important finding because some plant fractions showed high selectivity towards *P. falciparum.* The anti-inflammatory effect described for *A. pyrifolium* [45] might be useful in severe malaria treatment in which an aberrant inflammatory reaction results from the host immune response to the parasites with deleterious consequences like vascular activation and dysfunction [47]. In addition, an effective immunomodulatory therapy might improve clinical outcome and decrease long-term neurological sequelae [48].

Previous work with *A. pyrifolium* foliage or with the entire plant has demonstrated its toxicity to goats, sheep and cattle resulting in abortion, premature birth and damage to rat embryos [44, 49, 50]; the plant stem bark, fruit and roots were toxic to larvae of *Plutella xylostella* [51]. In addition, all such fractions (Table 1) showed low in vitro cytotoxicity. However, this toxicity was not observed in animals used in the Parkinson’s disease model [45]. Furthermore, the most promising fraction (AP5F.ALC) from the crude stem extract, based in the IC_50_ and SI values, showed no increased potential for mutagenicity and genotoxicity as judged by the Ames test.

The high selectivity index of *A. pyrifolium* was comparable to that of other species of *Aspidosperma* published before [29–31]. *Aspidosperma pyrifolium* was the second-best species for in vitro activity against malaria parasites, only preceded by *A. nitidum*, a species highly used as anti-malarial treatment in Brazilian malaria-endemic areas [29]. The two other species *Aspidosperma olivaceum* [30] and *Aspidosperma ramiflorum* [31] showed higher toxicities, resulting in lower selectivity indexes when compared to *A. pyrifolium.*

Experimental studies with artemisinin-derived drugs have shown regulation of the innate and adaptive immunity inflammatory reactions [52]. It has been well documented that a disproportionate body inflammatory response to blood stage parasites causes severe malaria, resulting in high morbidity during acute infections [53], indicating that the immune system plays an important role in limiting parasite density in malaria [54]. Whether the anti-inflammatory action of *A. pyrifolium* is responsible for its anti-malarial effect in rodent malaria is yet to be demonstrated.

**Fig. 4:**
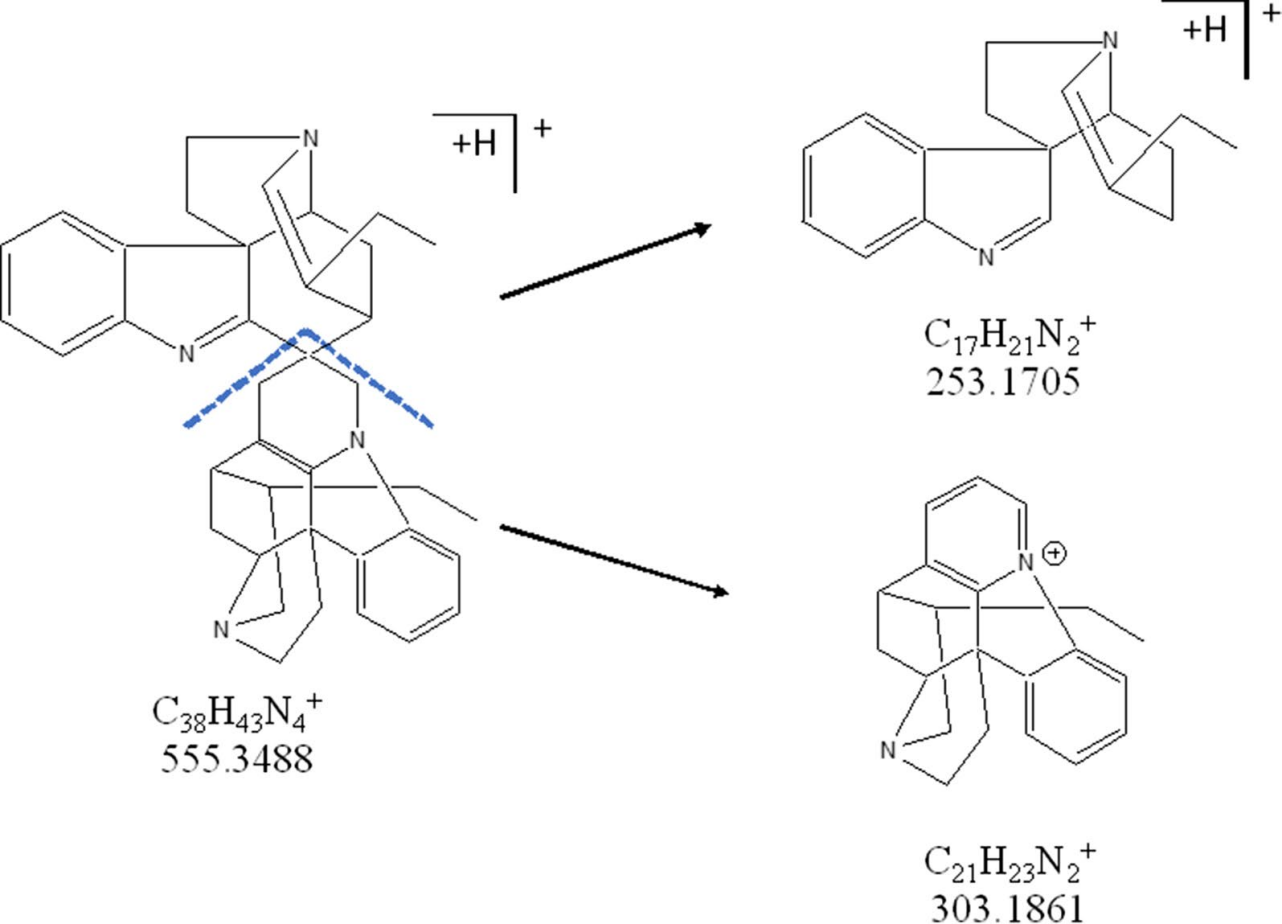
Putative fragmentation of compound A (Leucoridine B)

UPLC-HRMS analysis of AP5-FALC showed the presence of two major components in the active fraction, for which the molecular formulas C_38_H_42_N_4_ and C_41_H_46_N_4_O_2_, were proposed. A literature search in the Chemical Abstracts database looking for compounds with molecular formula C_38_H_42_N_4_ (compound A) retrieved only the bisindole alkaloid Leucoridine B, which was previously isolated from the stem-bark extract of *Leuconotis griffithii*, a species belonging to the Apocynaceae family [55]. Indeed, analysis of the HRMS-MS spectra of **A** (Figs. 3d and 4) showed a fragmentation pattern that is compatible with the Leucoridine B structure. The proposed fragmentation can explain the major signals detected in the MS2 spectra of this component of the mixture, namely the ions with *m/z* 303.1861 and 253.1405.

**Fig. 5:**
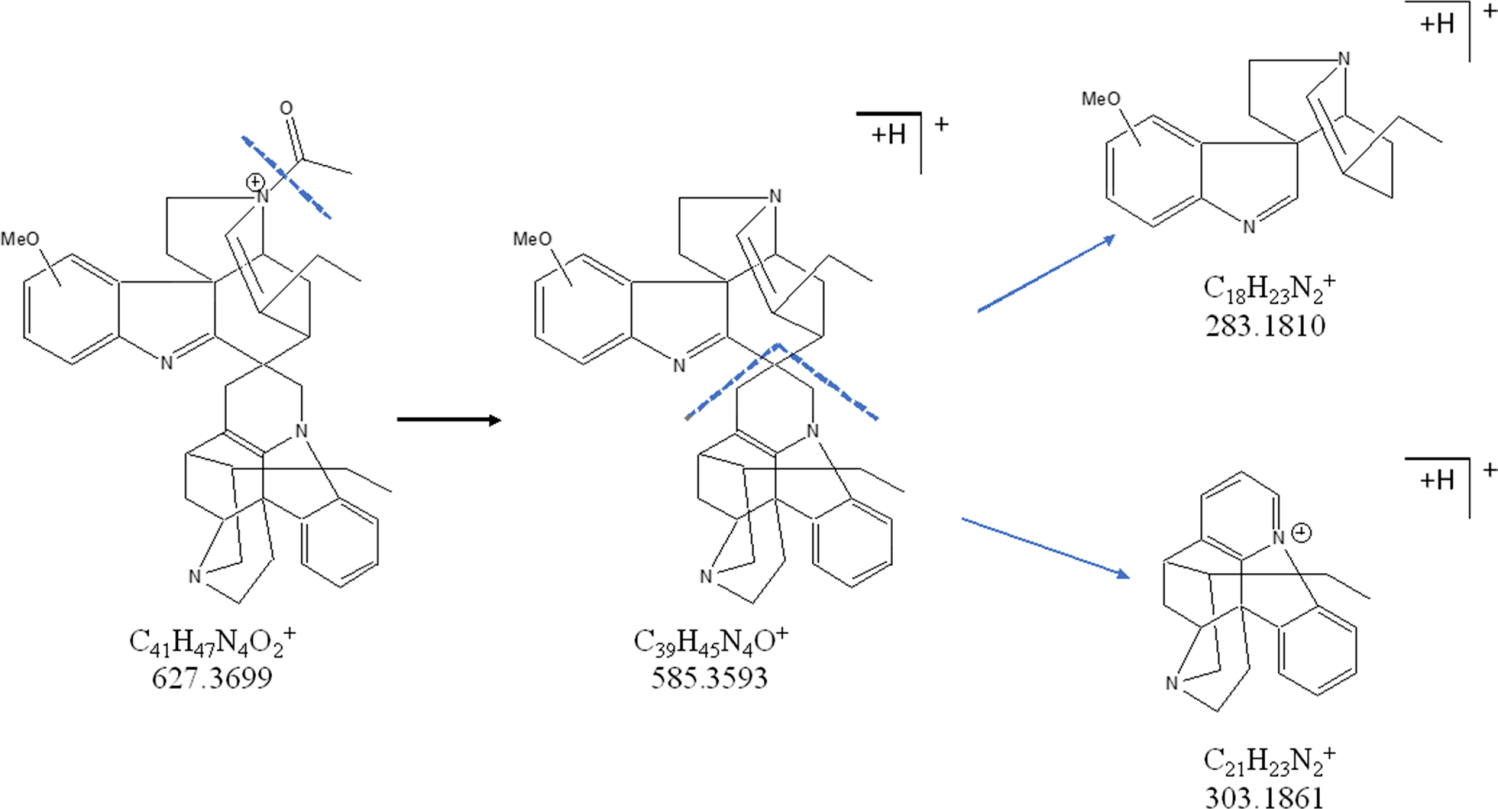
Putative fragmentation of the molecular ion for the proposed compound B (Leucoridine E). The methoxy and acetyl group positions are not defined

In contrast, no natural product with the formula C_41_H_46_N_4_O_2_ could be found, indicating that component **B** may be a novel compound. Its molecular formula and the presence of the fragment ion with *m/z* 303.1861 (Figs. 4 and 5) indicate that it may have a structure related to that of **A**. The observation of an ion with *m/z* 585.3593 was interpreted as a loss of a CH_2_CO moiety, probably from an acetylated nitrogen. The presence of a methoxy group in an aromatic ring sums up to the calculated formula for the ion C_41_H_47_N_4_O_2_^+^ with *m/z* 627.3698. To explain the ions with *m/z* 303.1861 and 283.1810, both new substituents must be on the same half of the bis-indole structure, explaining the ion with *m/z* 283.1830. Unfortunately, there was only 2 mg of AP5F.ALC left for this study and the confirmation of the proposed structures will require isolation of larger amounts of **A** and **B** for their unequivocal identification.

## Conclusion

The *A. pyrifolium* was the second-best *Aspidosperma* species for in vitro activity against malaria parasites, only preceded by *A. nitidum*, a species highly used as anti-malarial treatment in Brazilian malaria-endemic areas [29]. The two other species *A. olivaceum* [30] and *A. ramiflorum* [31] showed higher toxicities, resulting in lower selectivity indexes when compared to *A. pyrifolium*. This plant species is likely to be useful for further development of an anti-malarial drug, but supplementary chemical and pharmacological studies are still needed.
